# AURORA - An Automatic Robotic Platform for Materials Discovery

**DOI:** 10.1021/acsami.5c02605

**Published:** 2025-04-23

**Authors:** Bingyu Lei, Per H. Svensson, Pavel Yushmanov, Lars Kloo

**Affiliations:** † Applied Physical Chemistry, Department of Chemistry, KTH Royal Institute of Technology, Stockholm SE-114 28, Sweden; ‡ Oral Product Development, Pharmaceutical Technology & Development, Operations, AstraZeneca, Gothenburg SE-431 53, Sweden; § P&L Scientific AB, Lidingö SE-181 39, Sweden

**Keywords:** robotized screening, automatic platform, material discovery, perovskite-like materials, mesoscopic solar cells

## Abstract

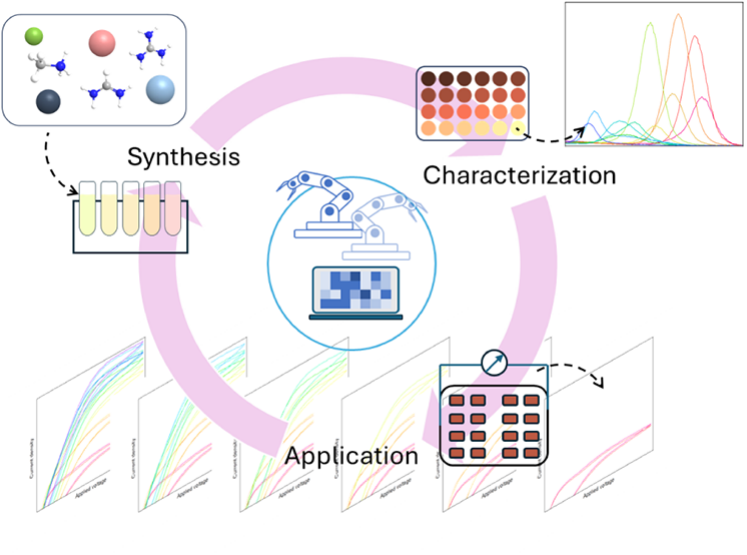

The urgent need for renewable energy solutions requires rapid advancements in materials discovery. In response, we present AURORA, an innovative robotic platform that enhances this process by integrating automated synthesis, characterization, and evaluation into a single unit, thereby improving efficiency and reducing errors. Its modular design allows for adaptable screening of diverse materials, including metal halide perovskites, and their application in solar cell devices. Our study demonstrates the ability of AURORA to autonomously synthesize and evaluate polycrystalline, mixed halide perovskites, including a novel mesoscopic solar cell array with improved data reliability and throughput. AURORA also conducts postsynthesis treatments and dynamic analyses under stress, setting it apart from traditional methods. These features make AURORA a transformative tool for the discovery of novel materials, with potential machine learning integration for optimization. Our results highlight the application of AURORA as a robust and adaptable platform for future developments in automated materials research.

## Introduction

The discovery and design of novel high-performance materials for specific applications present a formidable challenge due to the vast chemical space that encompasses numerous potential candidates. This complexity is amplified by the considerable time and resources required to systematically screen and evaluate all possible combinations. For example, metal-halide perovskites (MHPs), currently the most promising photovoltaic materials, adhere to a general formula ABX_3_ referring to their 3D family of compounds, where A is a monovalent cation that can be either organic, such as CH_3_NH_3_^+^ (MA^+^) and HC(NH_2_)_2_^+^ (FA^+^), or inorganic, like Cs^+^. B is typically a divalent p-block metal cation (e.g., Pb^2+^ and Sn^2+^), forming an inorganic framework of BX_6_ octahedra linked together via halide anion ligands, including I^–^, Br^–^, or Cl^–^.^1^ In addition, low-dimensional perovskite-like materials (forming 2D, 1D, and 0D structures) can be synthesized when other ions are introduced to the sites that cause the 3D network of BX_6_ octahedra to collapse into lower dimensions of sheets, chains, or even isolated complexes.^2−4^ Another strategy to expand the family of materials related to the 3D perovskites is to replace two divalent metal cations in the B position with one monovalent metal ion and one trivalent metal ion, resulting in a new group of double-perovskite-type materials with the formula A_2_B^I^B^III^X_6_.^5^ Already these small compositional modifications lead to a countless number of members in the library of MHP-based materials, not to mention the consequences of further expansion by doping or alloying those pure MHP-like compositions.

Screening a selected subgroup of materials can represent a huge challenge, characterized by repetitive and painstaking experimental work. For these reasons, an automated screening platform is highly desired and beneficial to reduce routine human labor and minimize errors thereby unlocking a straightforward strategy for systematic studies of material properties.^6,7^ At present, robotic platforms have been introduced to different areas of materials discovery, such as a robotic platform for the synthesis of colloidal nanocrystals,^8^ a mobile robot-based platform focusing on organic synthesis,^9^ an automated biomateriomics platform,^10^ and the Poseidon robotic platform for the discovery of lithium-ion battery electrolytes.^11^ There have also been several platforms developed specifically for MHPs and perovskite-related technologies.^12−14^ In this work, we introduce our new modular robotic system, AURORA, as a new member of the robotic platforms family. The system is designed as an automatic robotic platform for the screening of functional materials, which includes robotic synthesis, materials characterization, and evaluation with respect to a specific device application. MHPs have been selected as an illustrative case study. By combinatorial synthesis of mixed halide perovskite polycrystals, followed by photoluminescence investigation, the automatic fabrication of arrays of mesoscopic perovskite solar cells (mPSCs), and dynamic current–voltage (IV) performance characterization, we demonstrate the capability of the AURORA system for the screening of perovskite-like materials. The future development of the AURORA system into a self-driving platform for data collection, reflecting on the balance between quantity and quality in building reliable data sets for training new machine learning (ML) models, is also discussed.

## Results and Discussion

**Figure 1:**
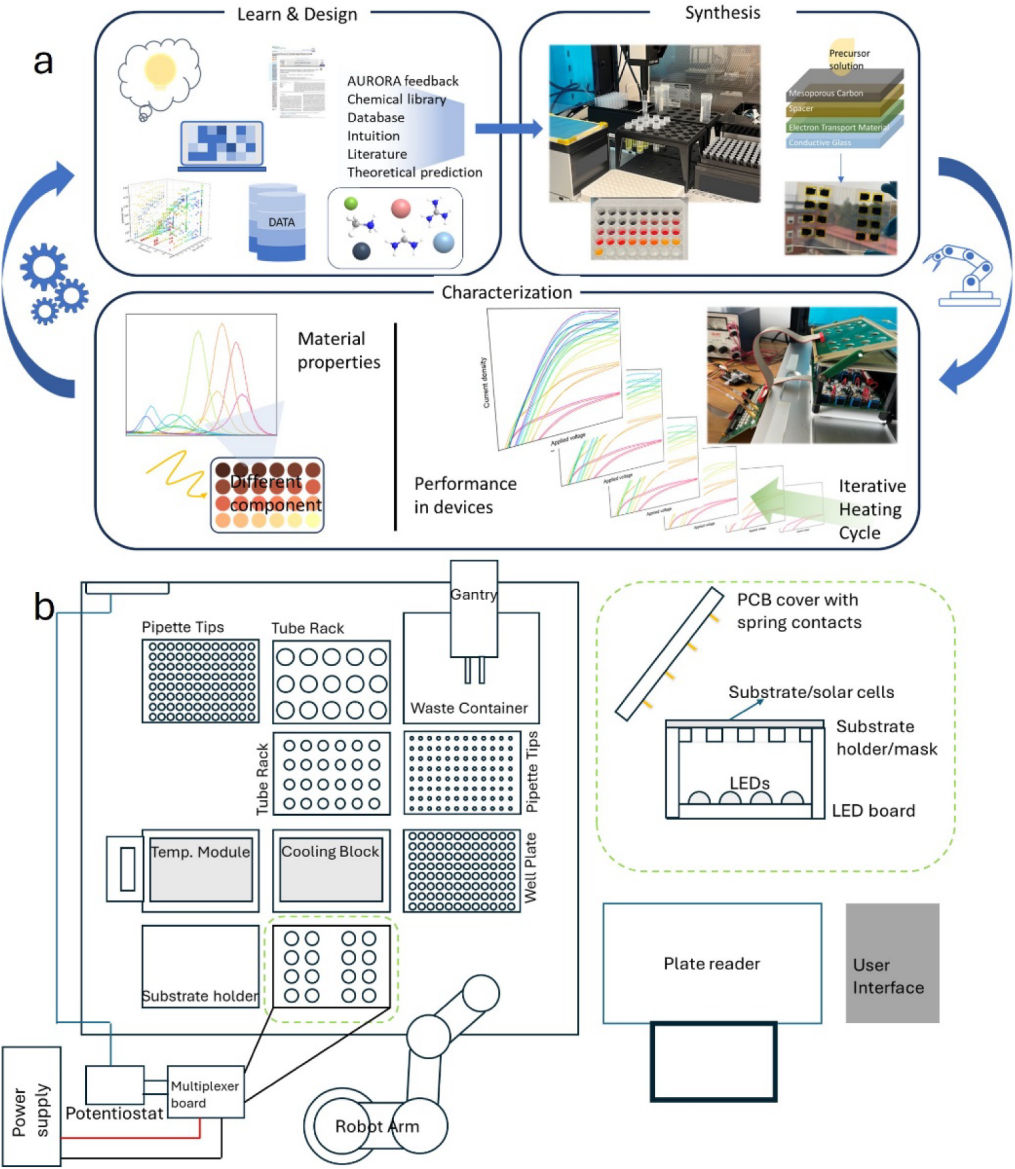
(a) A schematic diagram of the general framework for material development on the AURORA robotic platform and (b) the modular setup of the robotic system used.

The framework of the AURORA system is shown in Figure 1a. It was designed to perform synthesis, characterization, and application with ideas retrieved from existing systems in the literature, databases, and theoretically predicted results. The automated experiments provide possibilities to generate process-controlled data of high quality, which can offer feedback not only to the materials genome for future and more accurate predictions but also to the robotic system itself for self-optimization.

The AURORA system, used in this work for the MHPs case studies, consists of three fully integrated subunits: a robotic synthesis unit, a device test module, and a flexible robot arm. The robotic synthesis unit includes a liquid handling robot equipped with high-precision liquid handling labware (pipettes, tubes, and well-plates), a controllable temperature module, and a cooling stage. The robot arm can move samples between different units. In addition, there is a semiintegrated plate reader as a characterization module for photoluminescence spectroscopy (PL). An illustrative overview of the platform is given in Figure 1b.

The example MHP materials selected in this study are evaluated in mesoscopic solar cells. Such cells are sometimes referred to as monolithic solar cells, where mesoporous materials are precombined into a solar cell substrate, only missing the active photovoltaic material that can be simply added in the form of a precursor solution percolating through the top porous materials with subsequent solvent evaporation. The substrates used were based on a configuration of FTO/cTiO_2_/mTiO_2_/mZrO_2_/mCarbon; the prefix c relates to a compact layer and m to a mesoporous layer.^15^ This printable mesoscopic structure has proven useful in laboratory research of MHPs due to comparable conversion efficiencies with respect to the conventional type of PSCs, high stability, and unique advantages in terms of cost and scalability for industrialization.^16^ Moreover, this configuration allows solution deposition and target material crystallization in the preprinted inorganic scaffold to constitute the very last but crucial step of cell fabrication, which is highly compatible with the employment of a dispensing robot. In addition, mesoscopic cells are less dependent on the excellent film-forming properties of the new materials generated, which may be a limiting factor in the discovery of new materials. As demonstrated by previous work from our group, the mesoscopic PSCs have shown excellent compatibility for photovoltaic materials screening.^17^ In this work, we have designed a new printable mesoscopic substrate array (Figure S1), allowing the simultaneous screening of 16 devices on a single substrate, with the potential to further upscaling to 32 or 64 cells on a single substrate and the implementation of parallel analysis.

The robotic solar cell device test module was designed for the 16-cell substrate architecture. A custom-made multichannel potentiostat assembly (P&L Scientific AB) was designed to consist of a single-channel potentiostat acting as a master unit, a power supply, a substrate holder containing 16 apertures with a mask area of 0.126 cm^2^, and three printed circuit boards (PCBs): a multiplexer board, a light-emitting diode (LED) board, and a board with spring-loaded contacts. The 16 LEDs were calibrated (details are given in Figure S2) to 1000 lx, which corresponds to a power intensity of 250.8 μW cm^–2^,^18^ which is a commonly used light intensity when studying the application of a material in indoor photovoltaics. IV characterization of the device performance involves the key parameters: short-circuit photocurrent density (*J*_sc_), open-circuit voltage (*V*_oc_), fill factor (FF), and power conversion efficiency (PCE), which were extracted via a control Python script. It is worth noting that the performance of the devices under dim light not only highlights the potential of the studied materials for indoor applications but also provides a reasonable correlation with their performance under 1 Sun (AM 1.5G, 100 mW cm^–2^) illumination. Example comparisons are given in Figure S3.

**Figure 2:**
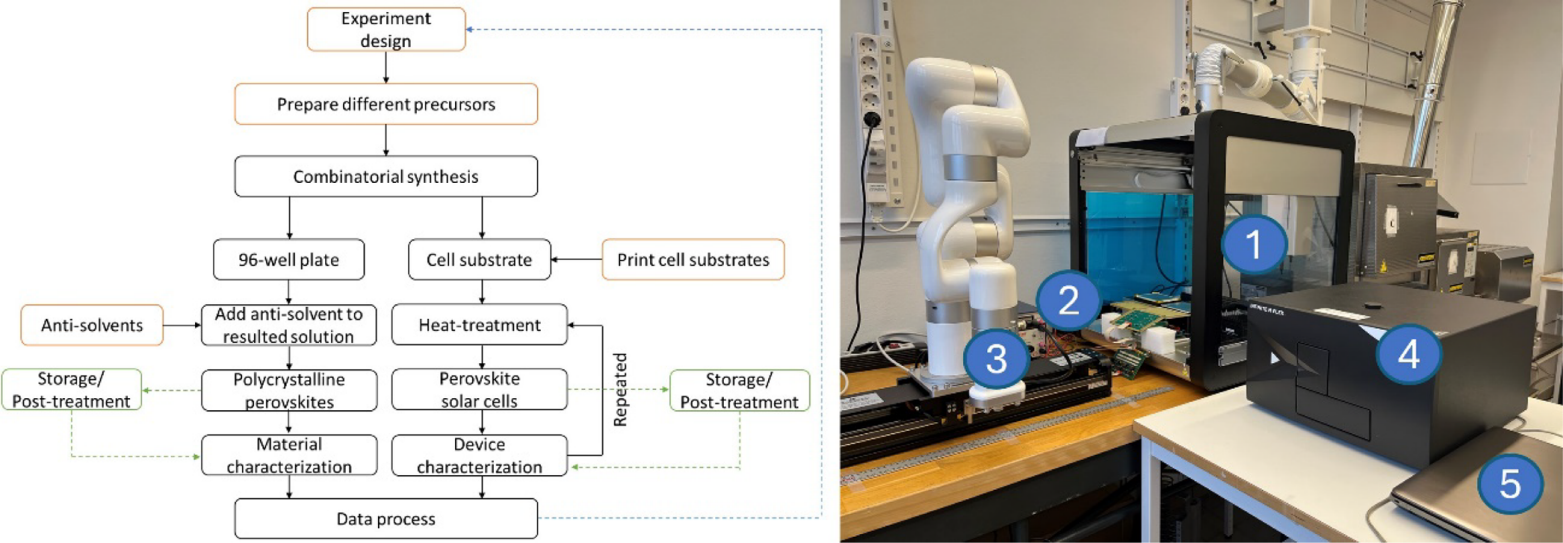
An illustrative workflow (left) and a photograph of the robotic platform (right), including (1) the dispensing robot, (2) the solar cell test module, (3) the robot arm, (4) the plate reader, and (5) the control laptop.

A proof-of-concept workflow and the corresponding units of the robotic platform are illustrated in Figure 2. The combinatorial synthesis was carried out in a liquid-handling unit. The resulting solutions were dispensed either into a 96-well plate for photoluminescence investigation or onto a preprinted mesoscopic inorganic scaffold array substrate of 16 solar cells. An antisolvent was added to the well plate for the precipitation of polycrystalline materials for evaluation. Following precipitation, the well plate is transported to the plate reader for characterization. In this work, we demonstrate the successful application of a workflow aiming at the synthesis of mixed halide perovskite materials with a new combination of solvent–antisolvent as compared to previous studies (Note S1).^19,20^ The PL results of mixed methylammonium lead triiodide (MAPbI_3_) in γ-valeroacetone (GVL) and methylammonium lead tribromide (MAPbBr_3_) in dimethylformamide (DMF) using acetic acid (AcOH) as the antisolvent are shown in Figure S4.

After precursor solution deposition, the solar cell substrates were transferred by the robot arm to the temperature module for solvent evaporation and crystallization. After programmed heating and cooling procedures, the robot arm transported the substrate to the cell test module for IV scans. The substrate can be programmed to be transferred between the temperature module and the IV-test module several times to repeat the heating-measurement cycle for dynamic monitoring of the device performance during the crystallization process.

This first example experiment is based on the well-known MAPbI_3_, one of the prototype MHPs. The fully automated workflow can be found in Note S2 and Video S1. Specifically, 16 solar cells were fabricated with 2 μL of MAPbI_3_ precursor solution dispensed on top of each cell. The heating temperature was set to 90 °C for 2 min, while the cooling temperature was 30 °C, with an additional 60 s on the cooling block. The heating–cooling characterization cycle was iterated 6 times, with two IV characterizations per cycle. Figure S5 shows the resulting 12 IV curves of the 16 solar cells. All 16 cells show similar performance with small deviations, indicating the reliability of the data acquired from the robotic platform. The variance of the performance parameters between the different cycles demonstrates the necessity of the iterative procedure applied, which enables dynamic monitoring of the performance parameters of the target materials. This dynamic evaluation allows for the identification of the optimal conditions for high performance, especially when there are differently screened components with unknown optimal crystallization conditions. This stage is also highly suitable for initial machine-learning implementation into the robotic system.

**Figure 3:**
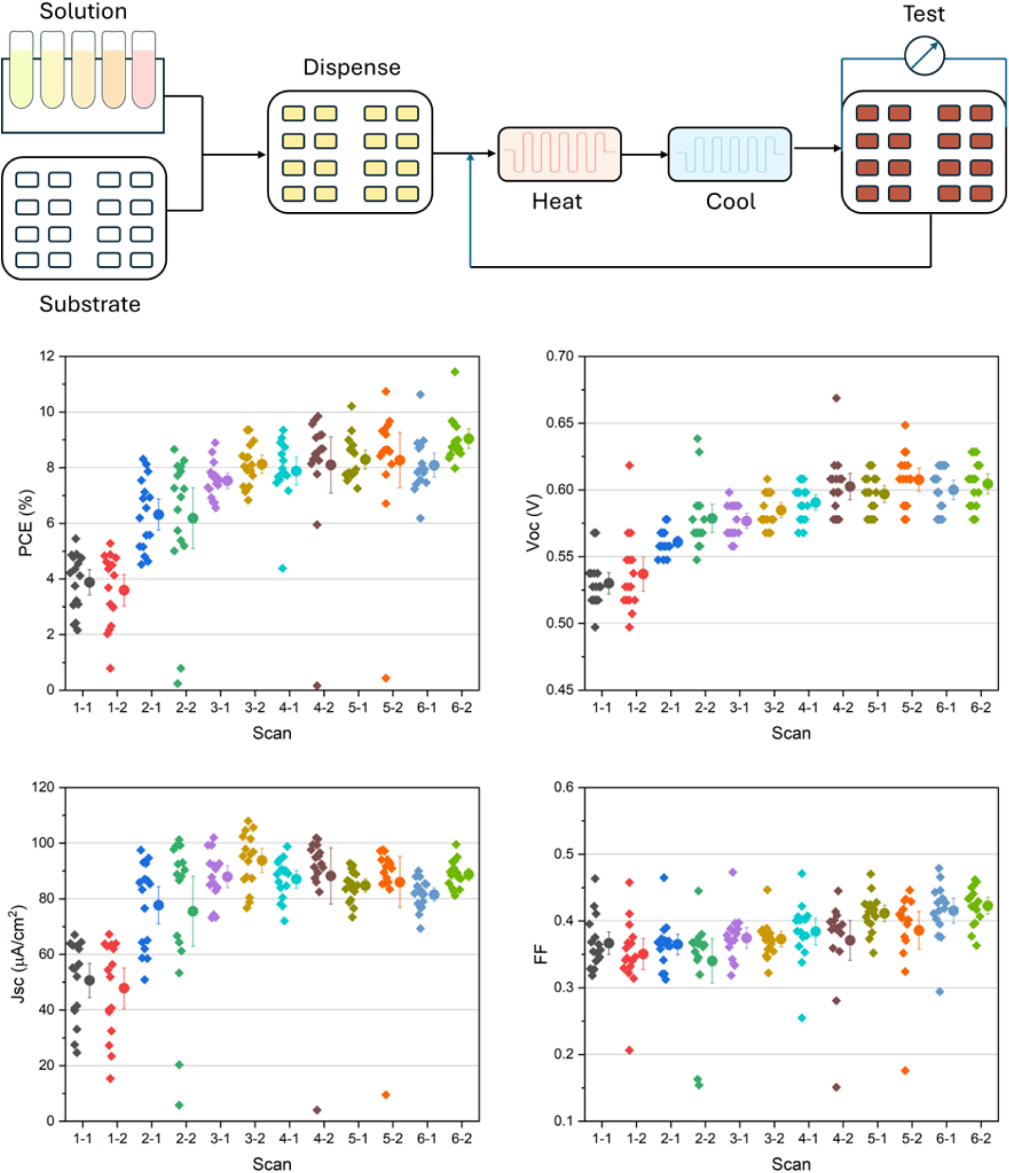
Top: schematic process of solar cell device fabrication and evaluation process. Bottom: extracted performance parameters from IV curves of the robot-fabricated MAPbI_3_-based solar cells. The plots show individual data points alongside the mean ±90% confidence interval.

From the IV-curves and extracted data, the reverse scan curves in some systems show nonideal shapes, leading to nonphysical, overestimated maximal power points, and fill factors. This phenomenon has frequently been reported for PSCs with carbon-based counter electrode materials, although the underlying reasons remain ambiguous.^21−23^ Therefore, the key parameters from forward scans were extracted for further discussion. As shown in Figure 3, there are obvious differences in the first 6 scans, i.e., three preparation cycles, especially regarding the increase in PCE, *V*_oc_, and *J*_sc_. This indicates that the conditions selected, involving a minimum of 3 preparation cycles, lead to a satisfactory degree of crystallization of the target material in the mesoscopic inorganic scaffolds. After 3 preparation cycles, the PCE and *V*_oc_ only increase marginally upon the introduction of more preparation cycles, while *J*_sc_ instead tends to decrease. The FFs of all investigated samples remain stable at around 0.4, with a gradual improvement as more preparatory cycles are applied. The relatively low FFs observed represent one trade-off required when an automated approach is used, which will be further discussed below. Overall, the standard deviation in the performance parameters for the different solar cells is within an acceptable range for screening purposes.

For comparison, one batch with 16 mesoscopic PSCs was manually fabricated using the same conditions as the robotized workflow, including the volume of solution, the heating temperature, set times, and cycles. However, the pipetting of precursor solutions and transfer of the substrates were carried out manually. The extracted parameters from the IV scans are shown in Figure S6. In general, the manually produced data show a trend similar to that of the robot-fabricated cells. However, the subtle variance in the pipetting and transfer by hand leads to different performance. Here, the high robustness and reproducibility of the robotized approach emerge as an additional and intentional benefit.

**Figure 4:**
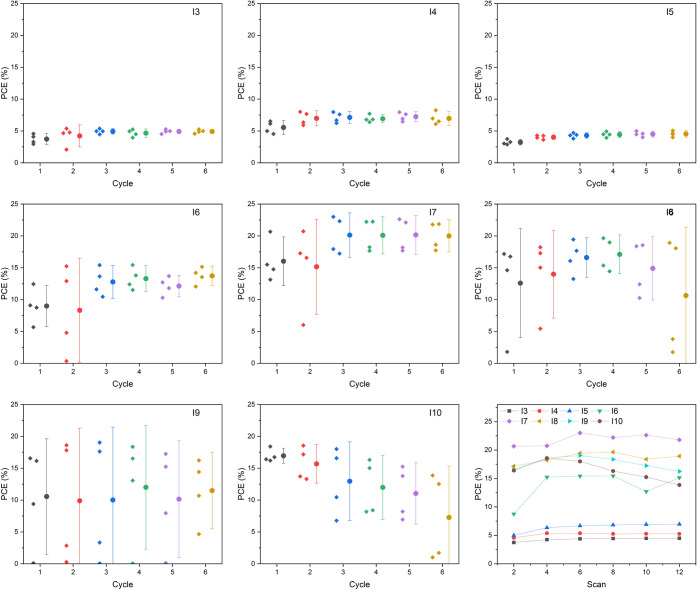
Extracted PCEs from forward IV scans of the robot-fabricated mixed halide MAPbX_3_ solar cells. The plots show individual data points alongside the mean ±90% confidence interval. The last figure offers a comparison compiled into one figure. For each condition, the second scan of each cycle and the cells without errors were selected for better visualization.

Mixed halide perovskite materials were also investigated in the robotic platform. Different compositions of the MAPbX_3_ (X = I, Br) precursor solutions were dispensed onto the 16 mesoscopic solar cell array. The percentage of iodide studied ranged from 30% to 100% in steps of 10% iodide (labeled as I3 to I10), and 2 cells were prepared for each composition. The heating–cooling characterization parameters were set to 90 °C for 5 min heating and 30 °C with an additional 60 s on the cooling stage, iterated for 6 cycles with 2 IV scans for each cycle. The resulting IV curves are shown in Figure S7, and the extracted forward PCE versus the cycle of iteration is shown in Figure 4. It is worth mentioning that, in our system, we focus on the heating–cooling iteration procedures and not a fixed heating time. As a consequence, 2 cycles of 5-min heating do not necessarily equal 5 cycles of 2-min heating. From the figures obtained, it can be deduced that the iodide-rich samples generally display higher PCEs than the bromide-rich samples under the selected cycling conditions. Overall, the MAPb(I_0.7_Br_0.3_)_3_ composition shows balanced performance and stability during the time of investigation, offering a highest PCE of 23.0% in cycle 3 and 21.8% in cycle 6, which may correspond to around 5% under 1 sun illumination according to the comparison in Figure S3. For better comparison and further improvement of the multicomponent MHPs, an adjustment of the workflow—especially regarding the heating–cooling-testing parameters—will be included in future studies.

**Figure 5:**
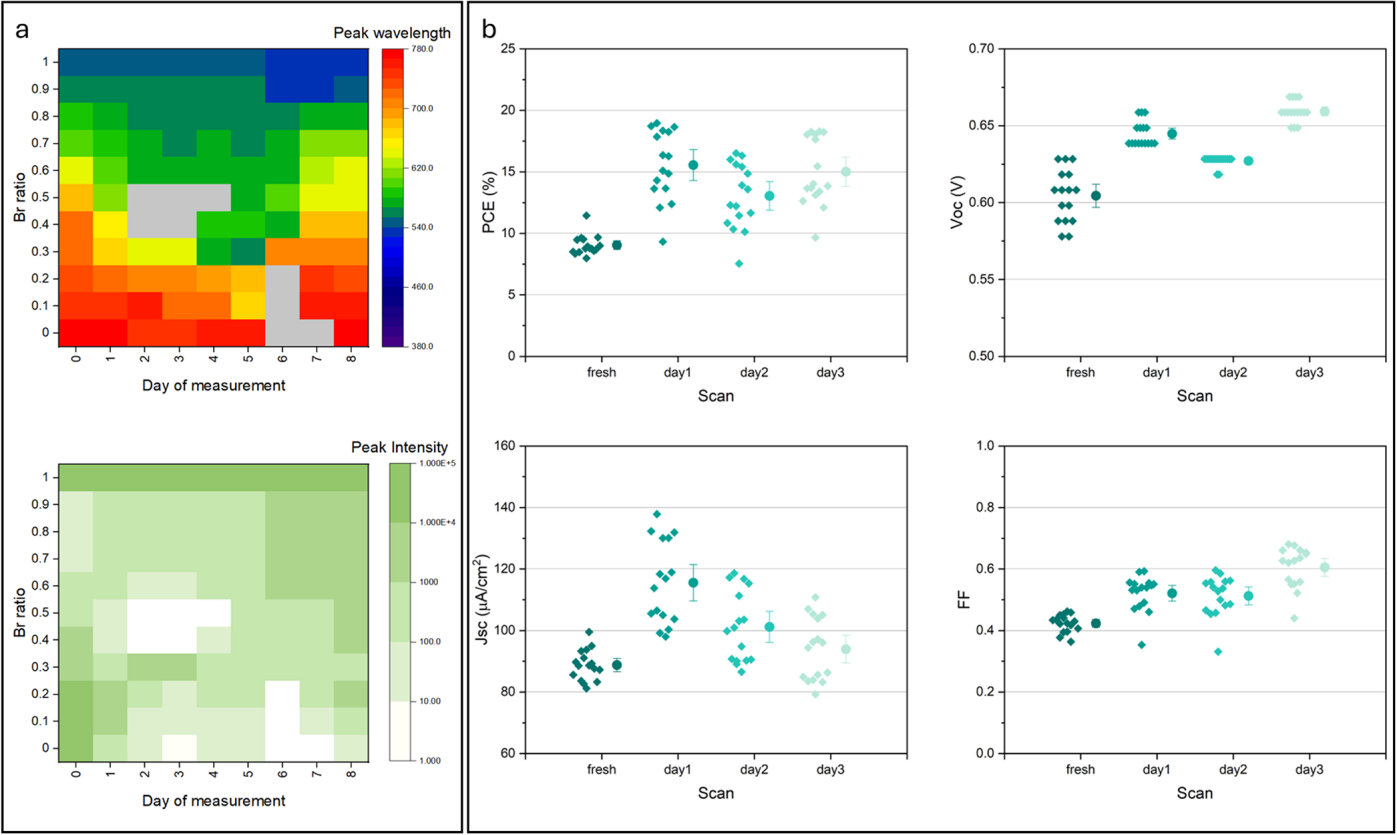
(a) Color maps of the peak wavelength and intensity variation of the PL spectroscopic results collected for the stored robot-synthesized samples. The gray blocks in the wavelength map and white blocks in the intensity map refer to spectra for which peak information cannot be extracted. (b) extracted parameters from forward IV-curves of the stored robot-fabricated solar cells. The plots show individual data points alongside the mean ±90% confidence interval.

In addition to the direct synthesis and subsequent analysis, the AURORA platform is also designed for post-treatments of the synthesized materials and devices, enabling dynamic analysis of the samples that are exposed to physical stress, chemical treatment, or simple on-the-shelf storage. In this study, the samples are transferred between storage and characterization modules. More complex post-treatments, such as heat treatment, light exposure, and solution treatment, will be included in future work. Figure S8 shows the resulting time-dependent PL spectra for the 11 synthesized MAPb(I_*x*_Br_1–*x*_)_3_ (*x* = 1 to 0) mixtures. The extracted peak wavelength and peak intensity are mapped against storage time to visualize the dynamic changes in the peaks during storage (Figure 5a). Generally, there is better stability in the bromide-rich samples, indicated by their smaller changes in peak wavelengths and intensities, which can be explained by the stronger interaction between Pb^2+^ and Br^–^.^24^ The storage investigation was also carried out for the robot-fabricated MAPbI_3_ devices. As shown in Figure 5b, all performance parameters improve after storage at ambient conditions (20 °C, ∼40% RH) for 1 day. Longer storage times under ambient conditions lead to degradation, as observed by a decrease in *J*_sc_. However, *V*_oc_ and FF continue to gradually increase, leading to a relatively stable PCE of the cells stored. This is consistent with published reports,^25^ and the observed behavior is explained by the competition between moisture-induced further crystallization and decomposition of the materials.

When designing a materials screening platform, there are inherent trade-offs, including throughput vs accuracy, speed vs depth, data quantity vs quality, versatility vs specialization, and automation vs flexibility. For example, the well-plate-based combinatorial synthesis followed by optical characterization has shown great potential for use in high-throughput experiments and for providing reliable optical properties of the screened materials. However, the automated procedure is not as accurate as a detailed manual study, which includes well-separated steps such as synthesis combined with structural analysis involving X-ray crystallography and electron microscopy, in terms of ensuring product structure and phase purity. The insights obtained from the static PL-spectroscopic evaluation are also limited compared to time-resolved techniques. This limitation also applies to the design of the device system in this work, including the use of mesoscopic solar cell arrays and the LED-based light source. It should be emphasized that our automated approach does not focus on the fabrication of a device with all technical parameters optimized but rather provides a fast way to understand the composition-structure-performance correlations of a target materials subgroup in devices such as solar cells.

For mesoscopic solar cells, very slow crystallization of the absorber in the inorganic scaffold benefits the device performance. In some reports, a cover is applied during the heating step to slow down the evaporation of the solvent used and, thus, the crystallization process.^26^ We tested this by manually fabricating 16 cells with MAPbI_3_ and heating them at 70 °C for 30 min using a cover (Figure S9). The average PCE from forward scans was (13.08 ± 3.36)%, with the best cell showing a reverse PCE of 22.6%; significantly better than a MAPbI_3_ solar cell heated on an “open” hot plate fabricated by the robot. However, this approach does not allow dynamic PCE monitoring and usually requires a significantly longer preparation time for better crystallization, which is not optimal for an automated process.

The versatility and flexibility should also be considered for a robotic platform. In the AURORA system, the materials characterization is currently more versatile than the device module, which has been designed for a specific type of solar cell configuration and measurement, although both still have limitations with respect to the scope of materials that can be studied. A high degree of automation is efficient for predefined workflows and provides more controlled and transferable parameters for reproducibility, avoiding human errors. However, it may also make the system less adaptable and less flexible. For this reason, we have designed the platform with reorganizable modules, which allow adaptation of the automation level according to the screening scenario and facilitate the implementation of new functional modules for materials or device characterization step-by-step. This also allows AURORA or each module used in this work to be easily integrated with other robotic materials platforms. It is notable that the core robotic modules offer significant capabilities with respect to cost, and the flexible expansion by implementation of new modules for characterization is facilitated by the large number of the current availability of relatively inexpensive table-top instruments.

In order to provide an overview of the current state of AURORA, we present a comparative table (Table S1) that highlights its existing capabilities, strengths, and areas for future improvement. For comparison, we have selected some robotic platforms that focus on MHPs, considering the model materials used in this work, although there are many other systems available, and these robotic platforms, including ours, may also be applicable to other types of materials.

In order to further improve AURORA and balance the aforementioned trade-offs of a screening platform, the next steps in robot extension will be to add modular powder X-ray diffraction and diffuse reflectance spectroscopy units. This approach is highly compatible with a hierarchical screening workflow and the use of hybrid screening methods. For example, a workflow can include prescreening of perovskite-inspired materials, starting with the solution-based synthesis in the current liquid-handling module and high-throughput photoluminescence analysis in the plate-reader module, followed by moderate-throughput phase identification for the selected promising candidates. Ultimately, the most promising materials can be studied in the device module. Modules for battery evaluation are currently under construction to expand the screening scope of materials relevant to renewable energy projects, such as electrolyte materials for Li-ion batteries.^11^ In addition, as mentioned before, the robotic platform is highly suitable for machine-learning implementation. For instance, a Bayesian optimization process integrated into the synthesis-characterization workflow can acquire the optimized device performance with minimal experimental trials for interesting materials/device candidates through a real-time feedback loop. It is also possible in the future to implement inverse design to identify optimal compositions for new materials based on the collected materials and/or device screening data. In order to achieve this, it is essential to further enhance the data reliability and standardize the workflow through the robotic platform.

## Conclusion

In summary, we have developed the AURORA robotic screening platform for materials discovery, demonstrated through initial experiments with the well-known MAPbX_3_ materials family (X = Br, I). Our proof-of-concept experiments highlight the capability of AURORA for perovskite materials synthesis, PL spectroscopic characterization, and dynamic performance evaluation, including the development of a novel mesoscopic solar cell array. The automation protocols reduce human effort and error in the data collection, enabling the creation of a reliable, high-quality database suitable for self-learning algorithms and facile data transfer to other users. Although still in an early phase of development, the AURORA system operates through an automated workflow that efficiently screens materials subfamilies. Looking ahead, by integrating feedback from current experiments and adding more modules of characterization, we envision expanding the system’s capabilities to explore a broader range of materials, unlocking new possibilities and applications. This will facilitate the evolution of AURORA into a robust, self-driving platform, ready to meet the dynamic challenges of modern materials research and discovery.

## Experimental Section

### Materials

Laser-patterned glass substrates with a conducting layer of fluorine-doped tin oxide (FTO) of 7 Ω sq–1 sheet resistance were purchased from Yingkou Shangneng Photoelectric Material Co., Ltd. Methylammonium iodide (MAI) and methylammonium bromide (MABr) were purchased from Greatcell Solar Ltd. Lead iodide and lead bromide were purchased from TCI Co. Titanium diisopropoxide bis(acetylacetonate) (Ti(acac)_2_OiPr_2_), *N*,*N*-dimethylformamide (DMF, anhydrous), dimethyl sulfoxide (DMSO, anhydrous), isopropanol (IPA, anhydrous), acetic acid (AcOH), and γ-valerolactone (GVL) were purchased from Merck. Isopropanol and acetone were purchased from VWR. TiO_2_ (T165), ZrO_2_, and carbon pastes were purchased from Solaronix. All reagents were used as received without further purification or treatment, unless otherwise stated.

### Instrumentation

The robotic system consists of the following components:

- Liquid handling robot: Opentrons OT-2 pipetting robot
- Robot arm: Ufactory Xarm 6 integrated with Ufactory vacuum gripper
- Plate reader: TECAN Infinite M Plex
- A custom-made multichannel potentiostat assembly was designed by P&L Scientific AB, Sweden. It consists of a single-channel EmStat4S potentiostat (PalmSens Inc.) acting as a master unit, a power supply, a substrate holder containing 16 apertures with a mask area of 0.126 cm^2^, and three printed circuit boards (PCBs): a multiplexer board, a light-emitting diode (LED) board, and a board with spring-loaded contacts. The multiplexer board is based on a 4-bit counter and 16 × 2 solid-state switches, which sequentially connects to each solar cell via the board with spring-loaded contacts and lights up the corresponding LED. The LEDs have independent current-adjustable LED drivers. The EmStat4S potentiostat is customized with two digital (TTL) trigger outputs extracted from its PCB board and acts as a master unit. These trigger outputs form TTL pulses programmed by the MethodSCRIPT (PalmSens Inc.) software, embedded in a Python script. The first trigger output resets the 4-bit counter before the start of the measurement sequence, and the second trigger output steps up the counter to the next number, providing the sequential on/off switching of the LEDs and the sequential recording of IV curves of the 16 solar cells, respectively.

A schematic illustration is given in Figure S10 to show the communication and control scheme for the instruments used in this work.

### Preparation of Solar Cell Substrate Array

Laser-patterned FTO glass substrate was cleaned by sonication sequentially in detergent, water, isopropanol, and acetone. To deposit compact TiO_2_, a 0.2 M Ti(acac)_2_OiPr_2_ solution in anhydrous IPA was filtered by a 200 nm PTFE syringe filter and then spray-coated onto the clean substrate at 450 °C. The airbrush was held on top of the substrate at a distance of 10–15 cm, moved from top-left to bottom-right following a serpentine path exposed by the metal masks, and then moved backward, which was repeated 3 times. Compressed air was used as the gas source with a 2-bar output pressure. TiO_2_, ZrO_2_ and carbon were screen-printed layer by layer. TiO_2_ (7 mm × 7 mm for each) and ZrO_2_ (8 mm × 8 mm for each) were deposited on the negative electrode side of the substrate, while carbon (6 mm × 10 mm for each) was deposited across the etched line to bridge the two electrode sides. The alignment of each layer is shown in Figure S1. After printing each layer, heat treatment was carried out. TiO_2_ was heated at 70 °C for 30 min and then at 500 °C for 30 min, while both ZrO_2_ and carbon were heated at 400 °C for 40 min. Each screen contains 16 open areas, enabling the printing of 16 inorganic scaffolds on a single substrate.

### Combinatorial Synthesis and Measurement

#### Preparation

0.3 M MAPbI_3_ solution was prepared by dissolving PbI_2_ and MAI in GVL, and 0.3 M MAPbBr_3_ solution was prepared by dissolving PbBr_2_ and MABr in DMF.

15 mL tubes containing MAPbI_3_ solution, MAPbBr_3_ solution, and acetic acid, respectively, were placed in the tube rack in slot 11. Small empty tubes (2 mL) were placed in the tube rack in slot 8. 1 mL and 20 μL pipette tips were placed in slots 10 and 9, respectively. A 96-well microplate was placed in slot 6.

#### Robotic Synthesis

The synthesis starts with dispensing different volumes of MAPbI_3_ solutions in small tubes. Afterward, different volumes of MAPbBr_3_ solution were dispensed into each tube, followed by a customized mixing procedure. More specifically, the pipette extracts 70% of the solution from 1 mm above the bottom of the tube and dispenses it back at a height of 15 mm, repeating this process 10 times. From tube [0] to tube [10], the volumes of each component added are listed in Table S2. After all combinatorial solutions were prepared, 10 μL of each solution was transferred to a well of the microplate. To each well, 100 μL of acetic acid was added afterward.

#### Measurement

PL measurement was performed with TECAN Infinite M Plex. The PL signal, excited by a laser (450 nm), is collected from 500 to 850 nm in 2 nm steps.

### Cell Fabrication and Measurement

#### Preparation

MAPbI_3_ solution was prepared in a 2 mL tube by dissolving 0.461 g of PbI_2_ and 0.159 g of MAI in a mixture of DMF (630 μL) and DMSO (70 μL). The tube was placed in a tube rack in slot 6 of the Opentrons OT-2 robot. The printed cell substrate, masked by Kapton masks around each cell, was placed in the solar cell test module in slot 2 with the PCB cover open. Temperature module and cooling block were set in slots 4 and 5, respectively.

For mixed halide perovskite solar cells, MAPbBr_3_ was also prepared by dissolving 0.367g of PbBr_2_ and 0.112 g of MABr in the mixture of DMF (630 μL) and DMSO (70 μL). The volumes of MAPbI_3_ and MAPbBr_3_ added into the small tubes to form different MAPbX_3_ precursor solutions are listed in Table S3. A detailed workflow for robotic solar-cell fabrication and characterization is provided in Note S2.

#### Measurements

Photocurrent–voltage (IV) scans were performed by the custom-made multichannel potentiostat and were automatically initiated by invoking an IV-measurement package, which was modified based on the MethodSCRIPTExample_Python package provided by PalmSens (https://github.com/PalmSens/MethodSCRIPT_Examples/tree/master/MethodSCRIPTExample_Python). The MethodScript was edited as described in the Instrumentation section. For MAPbI_3_ solar cells, the scan started from −800 mV to 0, then back to −800 mV with a step size of 10 mV and a scan rate of 100 mV/s. For mixed halide perovskite solar cells, the applied voltage range was set between −1000 mV and 0, with other parameters unchanged.

## Supporting Information Available

Supplementary notes about the combinatorial synthesis and detailed workflow for solar cell fabrication and measurement; supplementary figures including the design and calibration of the solar cell substrate and test module, PL spectra, supplementary IV curves and extracted IV parameters; and supplementary tables comparing AURORA with selected similar platforms and showing details of combinatorial synthesis (PDF)
Supplementary video showing the workflows (MP4)
