# Supplementary material for: AURORA - An Automatic Robotic Platform for Materials Discovery
Source: ACS Appl Mater Interfaces. 2025 Apr 23;17(18):26701–9. doi: 10.1021/acsami.5c02605 (PMC12067375; doi:10.1021/acsami.5c02605)
Supplement: Supplementary file 1 — am5c02605_si_001.pdf [file am5c02605_si_001.pdf]

Supporting Information

AURORA - an AUtomatic RObotic platfoRm for mAterials discovery

Bingyu Lei[1], Per H. Svensson[2], Pavel Yushmanov[3], Lars Kloo[1,]*

[1]Applied Physical Chemistry, Department of Chemistry, KTH Royal Institute of Technology, Stockholm, SE-114 28 Stockholm, Sweden

[2]Oral Product Development, Pharmaceutical Technology & Development, Operations, AstraZeneca, SE-431 53 Gothenburg, Sweden

[3]P&L Scientific AB, SE-181 39, Lidingö, Sweden

Correspondence Email: lakloo@kth.se

Supplementary Note 1

Combinatorial synthesis is one of the most used methods for materials discovery due to the possibility of automation and parallelization. In the area of MHPs, it has been applied to construct sub-libraries within this wide scope of materials.[1,2] In many cases, it was combined with materials characterization, including absorption and/or PL spectroscopy and X-ray diffraction to map the relationships between composition, structure and property of the candidate materials. In this study, we adopt this broadly tested and feasible strategy into our automated system. The control program can be set to either scan linearly, as a pre-screening procedure, or perform parallel studies of the device characterization with a goal to promote the development of a results database including comprehensive parameters.

In this work we demonstrate the successful application of a workflow aiming at the synthesis of mixed halide perovskite materials. Even though this is an approach that has been applied in many previous studies,[1,3] our purpose is to demonstrate the feasibility of incorporating this strategy into our robotic platform. The example workflow includes: 1) mix different precursors in different proportions, 2) transfer the mixed solutions into the 96-well plate substrate for spectroscopic characterization, 3) add anti-solvent in each well to precipitate polycrystalline particles, 4) and record PL spectra in a plate reader. The results of mixed methylammonium lead triiodide ($MAPbI_3$) in γ-valeroacetone (GVL) and methylammonium lead tribromide ($MAPbBr_3$) in dimethylformamide (DMF) using acetic acid (AcOH) as anti-solvent are shown in Figure S4. The discussion on the selected chemistry is not the focus of this paper. However, it is still appropriate to mention that the mixture of iodide and bromide brings a change in the band gap of the $MAPbX_3$ (X=I, Br) solid solution system. It is also worth mentioning that a new combination of solvent-antisolvent was employed in this work. Because of this, the PL peak characteristics may differ slightly in comparison with published results.

Supplementary Note 2

Solar cell fabrication and measurement was controlled a python protocol uploaded to Opentrons software. Details for each step is shown below:

1) The robot arm transports the empty substrate to the cooling/dispensing stage.

Example code: `arm.set_servo_angle(angle=[97.2, 45.2, -102.9, 1.3, 58.0, 5.6], speed=5, wait=True, radius=-1.0)`

2) The liquid handling robot dispenses 2 μL of the synthesized/prepared solutions to specific sets of coordinates on the empty substrate. The pipette tip was calibrated to dispense at the centre of each TiO_2, 1 mm above the surface, which allows the surface tension and capillary force helping the thorough pull-out of such small amount of liquid.

Example code: `left_pipette.transfer(2, tube2.wells()[0], platedispens.wells_by_name()['A1'].top(69).move(Point(x=7, y=-1, z=0)), air_gap=1, blow_out=True, blowout_location="destination well", new_tip='never')`

3) The temperature module heats to the set temperature.

4) The robot arm transfers the substrate with dispensed precursor solution to the temperature module.

5) After the set heating time, the temperature module cools the substrate (i.e. the completed solar cells) to the set cooling temperature.

6) The robot arm then transfers the substrate to the cooling/dispensing stage for an additional cooling to ensure that the substrate has been cooled to room temperature before IV characterization.

7) The robot arm transfers the cooled substrate to the solar cell test module and covers it by the PCB with spring-loaded contacts.

8) the arm presses the PCB to ensure proper contact between the spring contacts and the electrodes of the solar cells.

9) The script invokes a solar cell test package to start the IV measurement and collect IV data for each solar cell sequentially.

10) After the measurement, the robot arm lifts and represses the PCB to start another measurement if more than one IV cycle was set to identify connection errors and to improve the data statistics. Otherwise, the robot arm opens the PCB cover to complete one heating-cooling-testing cycle (step 3 to 9).

11) If more than one heating-cooling-testing cycle is set, the protocol repeats step 3 to 10. Otherwise, the cell substrate is transferred to the substrate holder and the arm returns to its standby position to conclude the protocol.

Supplementary Figures

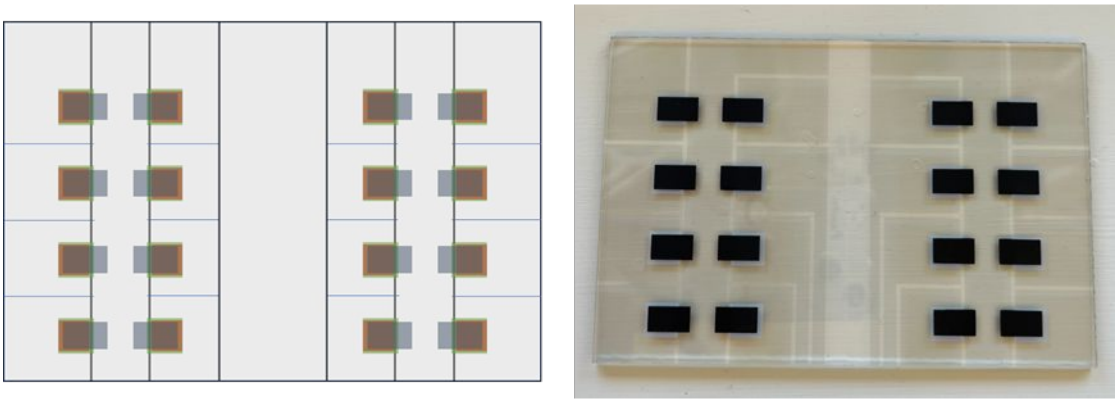

Figure S1 Schematic diagram (left) and a digital image (right) of the printable mesoscopic substrate array

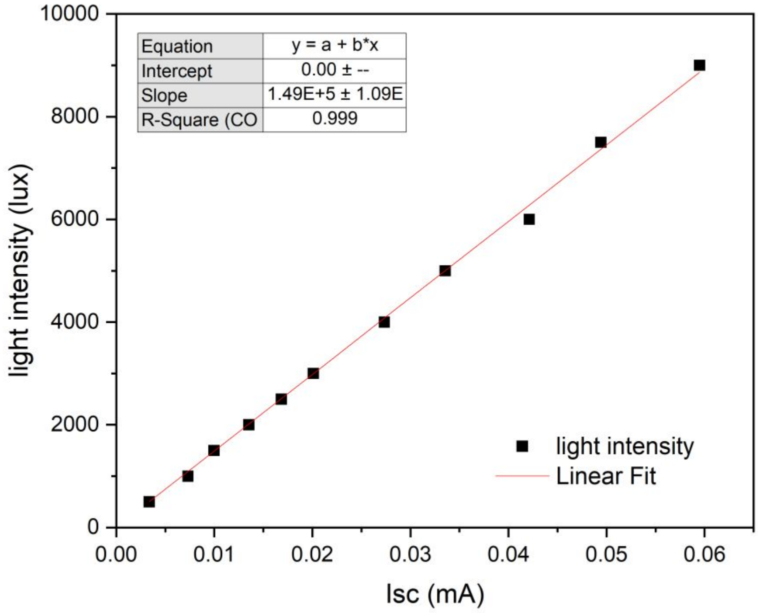

Figure S2 calibration curve of LEDs.

The calibration curve was generated by measuring the short-circuit current (I_{sc}) of a silicon diode under varying LED light intensities. The light intensity was adjusted and measured using an intensity meter. Based on the calibration curve, an intensity of 1000 lux corresponds to a short-circuit current (I_{sc}) of 6.71 μA. During the calibration of the LEDs in the solar cell test module, the same silicon diode was positioned at the location designated for each solar cell.

The independent current driver was adjusted for each testing coordinate until the I_{sc} reached 6.71 μA.

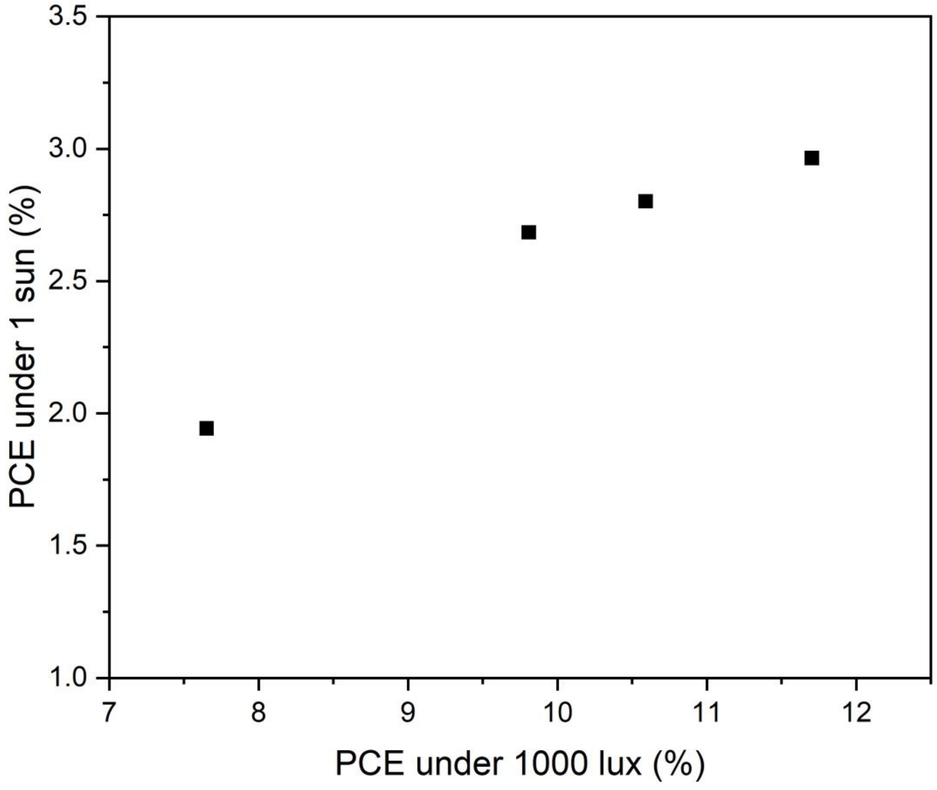

Figure S3 The comparison of the extracted PCE from the IV-measurement of selected cells under different light sources.

The cells were fabricated manually and individually. 1.5 μL $MAPbI_3$ solution was dropped on each cell followed by heating on 90 ˚C for 5 minutes. For 1 sun measurement, solar simulator was used as the light source and calibrated to 100 mW /cm^2 by a silicon diode. For LED measurement, the white LED was calibrated to 1000 lux by a light intensity meter. Both measurements were carried out on the same day as the cell preparation to minimize the impact of storage effects.

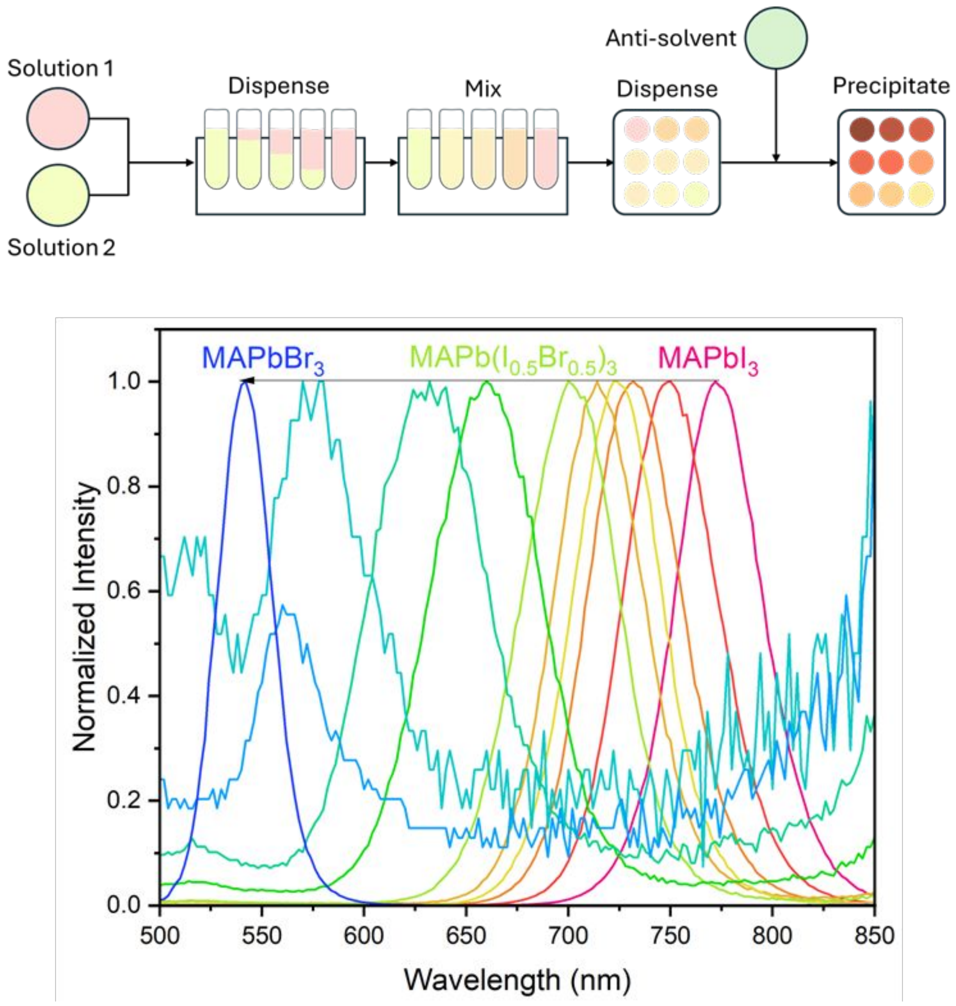

Figure S4 Top: schematic process of the combinatorial synthesis. Bottom: normalized PL spectra of the synthesized samples

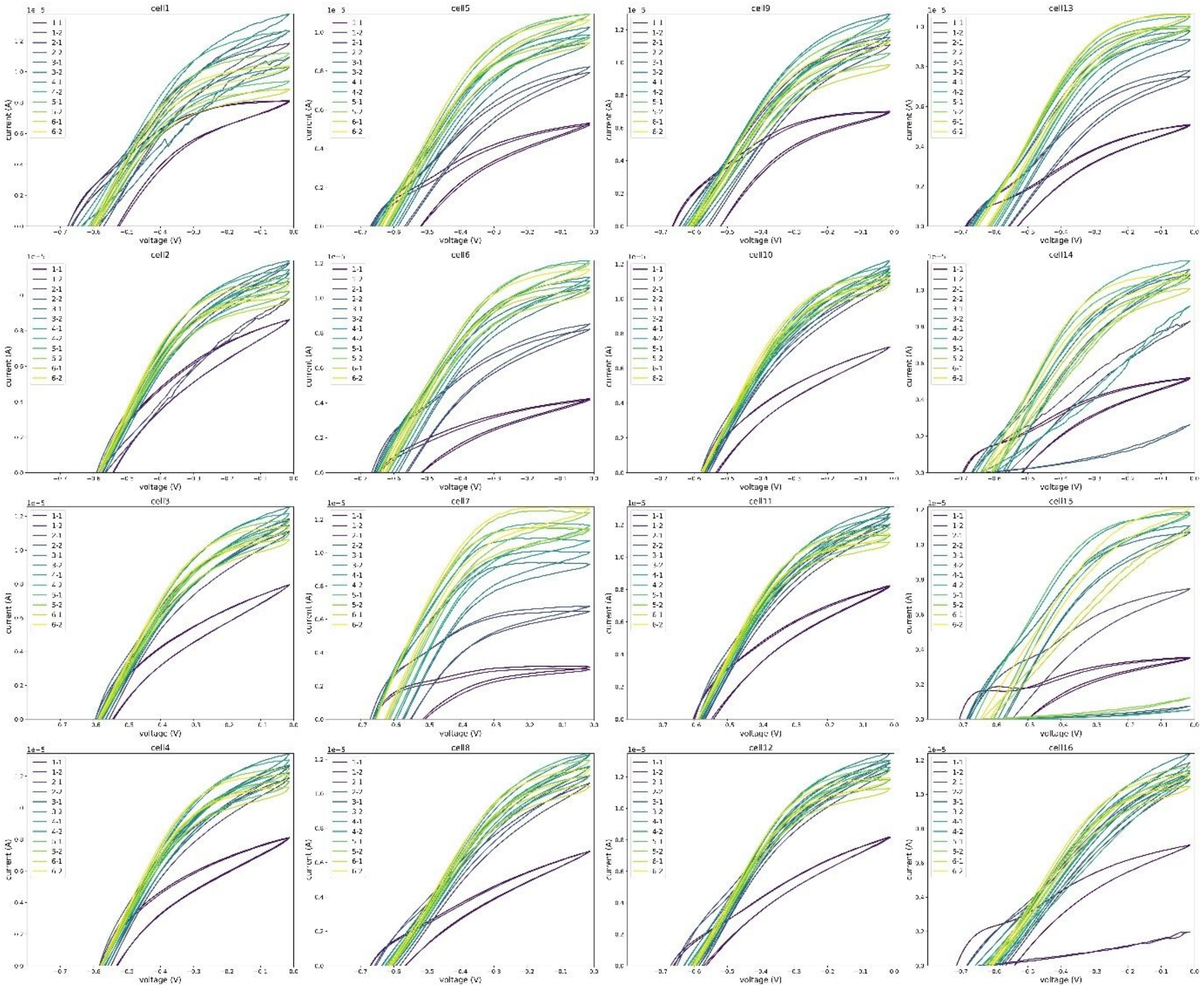

Figure S5 IV curves of the robot fabricated $MAPbI_3$ solar cells. The heating-cooling-testing parameters were set to 90˚C for 2 min, followed by cooling to 30˚C with an additional 60 seconds at the cooling stage. This process was repeated for 6 cycles with 2 IV scans conducted during each cycle.

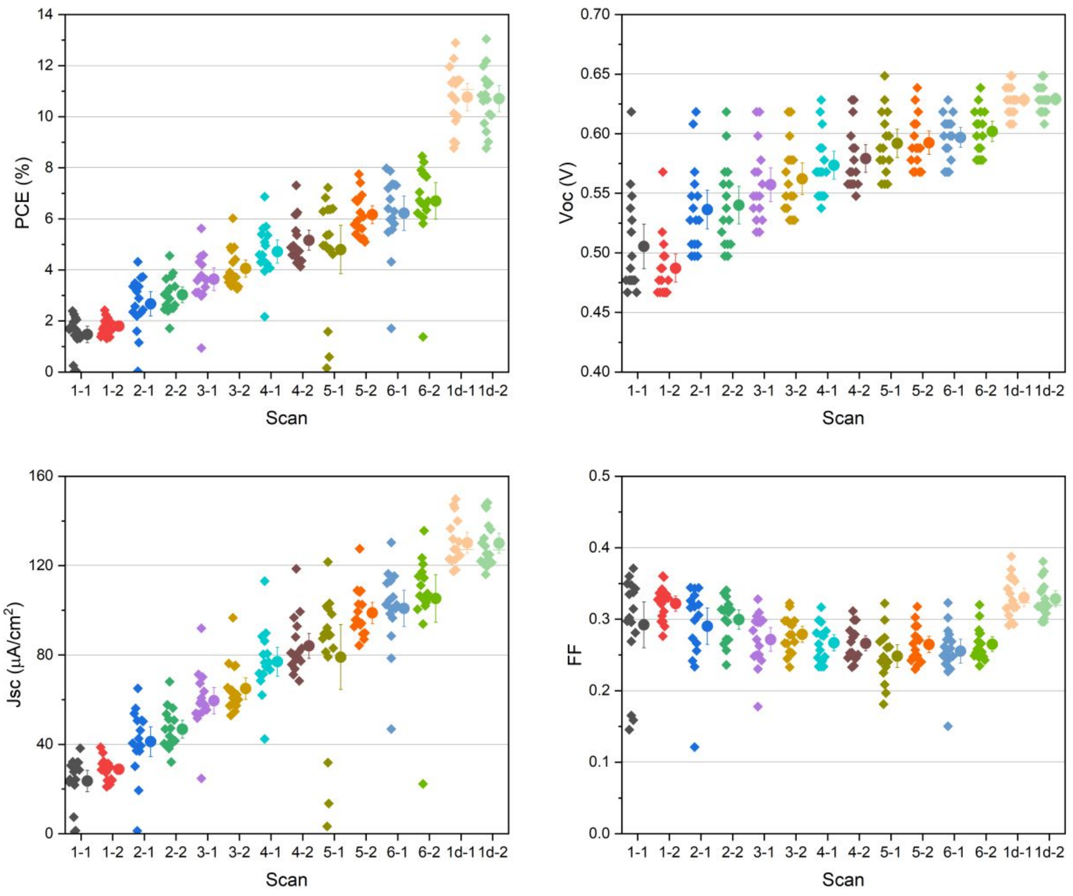

Figure S6 Extracted parameters from IV curves of the manually fabricated solar cells. 1d means the cells were measured after 1 day of storage. The plots show individual data points alongside the mean ± 90% confidence interval.

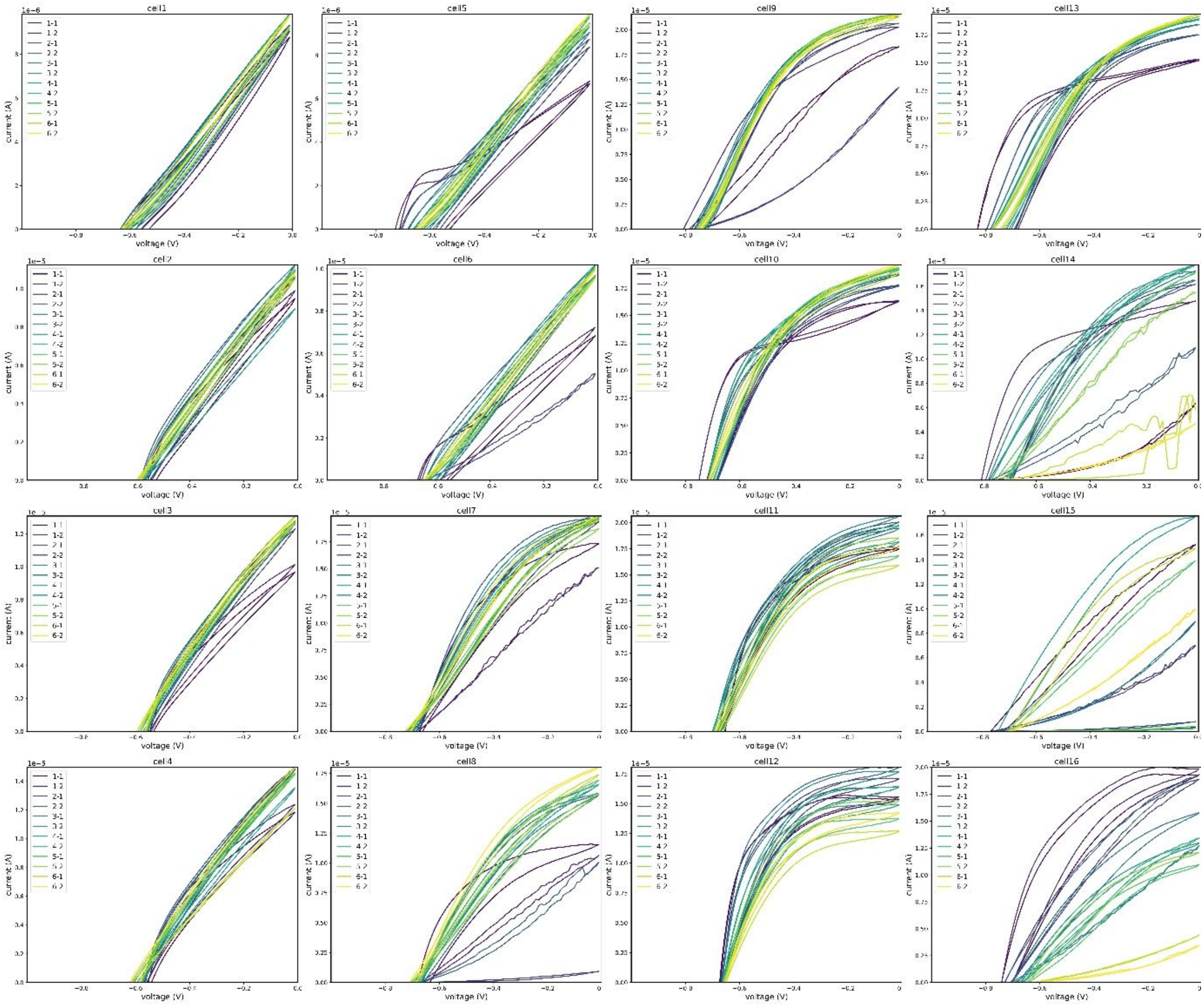

Figure S7 I-V curves of the robot fabricated $MAPbX_3$ (X=I, Br) solar cells. The heating-cooling-testing parameters were set to 90˚C for 5 min, followed by cooling to 30˚C with an additional 60 seconds at the cooling stage. This process was repeated for 6 cycles with 2 IV scans conducted during each cycle.

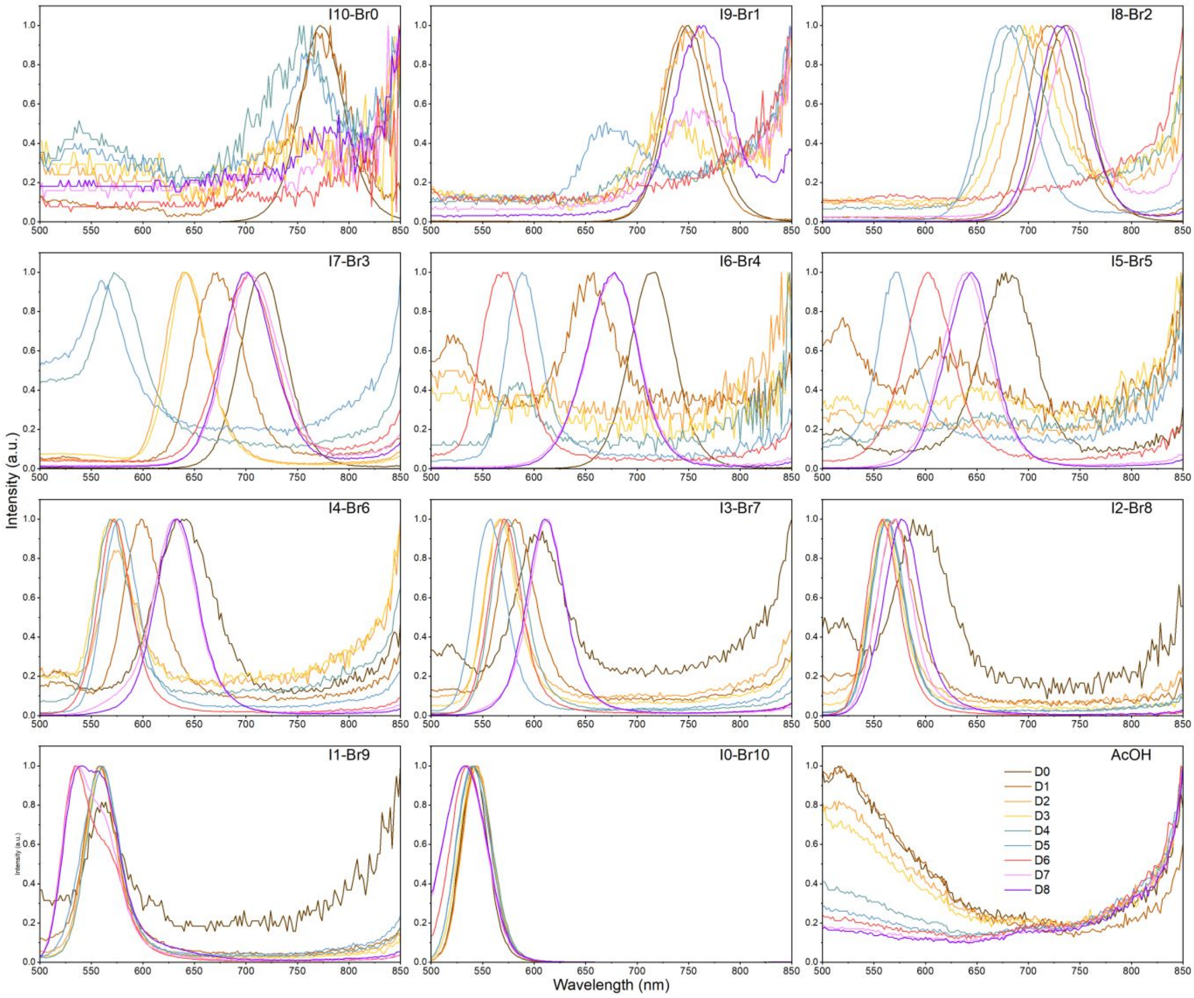

Figure S8 Normalized PL spectra of robot synthesized samples after different storage time.

The iodide-rich samples display a gradual shift in the PL-peaks to lower wavelengths and with a reduction in intensity. In addition, after 7-8 days of storage, new peaks emerge at short wavelengths gradually shifted back close to the initial positions. The bromide-rich materials also follow the same trend, but the changes become smaller the higher the Br percentage.

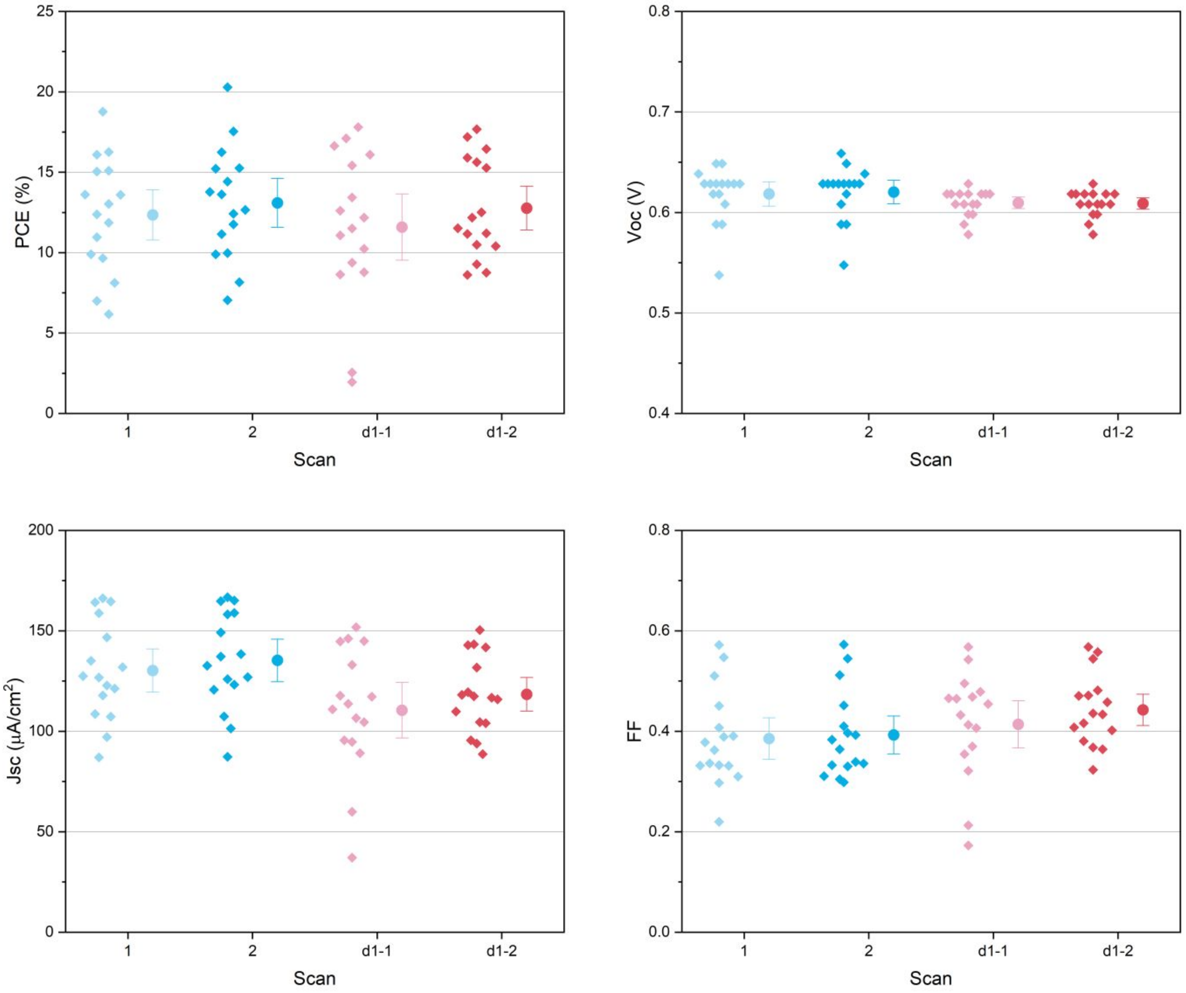

Figure S9 Extracted performance parameters from forward scans of manually fabricated cells. The solar cells were heated to 70 ˚C for 30 min under a cover to retard solvent evaporation and thereby the crystallisation process. The plots show individual data points alongside the mean ± 90% confidence interval.

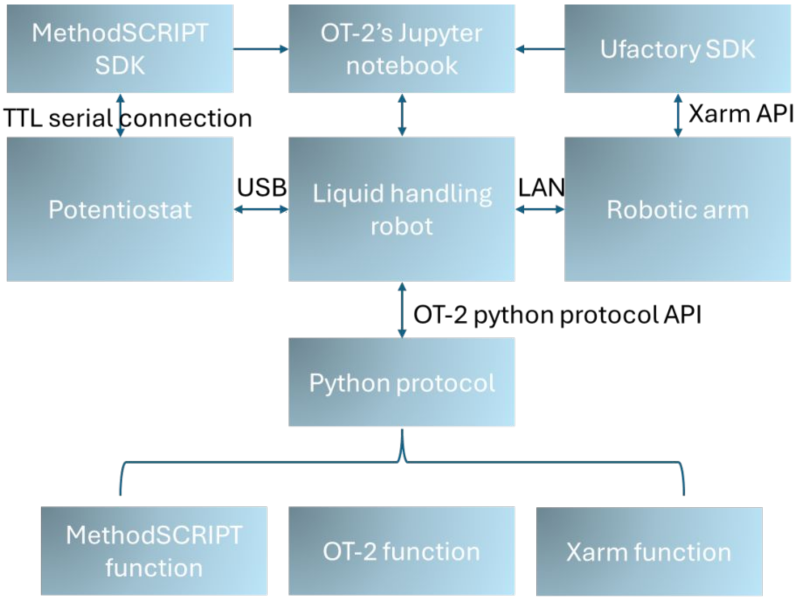

Figure S10 Schematic illustration showing the control and communication scheme used in this work.

Supplementary Tables

Table S1 Key features of AURORA and selected similar robotic platforms.

	AURORA (current stage)	PASCAL	SDFL	RoboMapper	AMADAP	MAOSIC
Overview	Automatic robotic platform for materials discovery including composition screening, characterization, automatic device processing and evaluation	Perovskite automated spin coat assembly line accelerates composition screening	A self-driving fluidic lab for autonomous nanomanufacturing	An automated workstation that can formulate and palletize semiconductors on a chip	Toward autonomous material and device acceleration platforms for emerging photovoltaics technologies	Automation platforms well integrated into a cloud lab
Synthesis Method	Automatic combinatorial synthesis and drop-casting	Automatic combinatorial synthesis and spin-coating	Automatic fluid delivery, mixing and reaction	Combinatorial synthesis by formulation bot and drop-printing on a chip	Robot-based pipetting, automated spin-coating	Automated microfluidic synthesis
Characterization Techniques	Photoluminescence (PL)	Integrated images and spectroscopy (PL and transmission) measurement	In-situ characterization module to automatically acquire the absorption and PL spectra of the in-flow synthesized NCs.	Wide-angle X-ray scattering, PL	Photography, UV–vis absorption, PL	In-situ absorption measurement, integrated chirality determination
Device Fabrication Method	Automatic drop-casting in custom inorganic mesoscopic scaffold	Perovskite films were fabricated by PASCAL, followed by	/	/	Active layer for conventional type of perovskite solar cells can	/

		off-line physical vapor deposition			be produced and optimized by SPINBOT[4]	
Device Evaluation Method	Automatic current-voltage measurement with custom potentiostat	Automated illuminated current-voltage testing	/	/	Current density versus voltage measurements	/
Target/Model Material	Polycrystalline metal halide perovskite	Multicomponent perovskite thin film	$Cs_3Cu_2I_5$ nanocrystals	Multicomponent perovskite pallets	Multicomponent perovskites, quasi-2D perovskites	$CsPbBr_3$ nanocrystal
Environment Control	Ambient condition recorded by an environment meter	Controlled glovebox environment	Precursors were prepared under N_2	A dry lab with controlled humidity	Different operational atmosphere	Equipped with environment sensor
Robot control	Python protocol	Python	LabVIEW, Python	LabVIEW	Hybrid control methods including graphical user interface	A software package implemented in Python
Human-in-Loop	Transfer PL samples and selective manual experiments for workflow evaluation	Transfer sub-cells for contact layers deposition and supportive thin film characterization	The system emphasizes autonomous operation	Transfer sample between synthesis workstation and characterization station. Supportive material evaluation	Complete device fabrication, communicate between platforms	Remote experimental design was enabled
Reference	This work	[5]	[6]	[7]	[8]	[9]

Table S2 Volumes of each component added to each tube during the robotic combinatorial synthesis.

Tube	0	1	2	3	4	5	6	7	8	9	10
$MAPbI_3$ (μL)	200	900	800	700	600	500	400	300	200	100	0
$MAPbBr_3$ (μL)	0	100	200	300	400	500	600	700	800	900	200

Table S3 The volumes of each component added to form different $MAPbX_3$ precursor solutions for the robotic fabrication of mixed halide perovskite solar cells.

Tube	0	1	2	3	4	5	6	7
$MAPbI_3$ (μL)	100	90	80	70	60	50	40	30
$MAPbBr_3$ (μL)	0	10	20	30	40	50	60	70

Supplementary References

(1) Higgins, K.; Ziatdinov, M.; Kalinin, S. V.; Ahmadi, M. High-Throughput Study of Antisolvents on the Stability of Multicomponent Metal Halide Perovskites through Robotics-Based Synthesis and Machine Learning Approaches. *J. Am. Chem. Soc.* **2021**, *143* (47), 19945–19955. https://doi.org/10.1021/jacs.1c10045.

(2) Lai, M.; Shin, D.; Jibril, L.; Mirkin, C. A. Combinatorial Synthesis and Screening of Mixed Halide Perovskite Megalibraries. *J. Am. Chem. Soc.* **2022**, *144* (30), 13823–13830. https://doi.org/10.1021/jacs.2c05082.

(3) Chen, S.; Hou, Y.; Chen, H.; Tang, X.; Langner, S.; Li, N.; Stubhan, T.; Levchuk, I.; Gu, E.; Osvet, A.; Brabec, C. J. Exploring the Stability of Novel Wide Bandgap Perovskites by a Robot Based High Throughput Approach. *Advanced Energy Materials* **2018**, *8* (6), 1701543. https://doi.org/10.1002/aenm.201701543.

(4) Zhang, J.; Le Corre, V. M.; Wu, J.; Du, T.; Osterrieder, T.; Zhang, K.; Zhang, H.; Lüer, L.; Hauch, J.; Brabec, C. J. Autonomous Optimization of Air-Processed Perovskite Solar Cell in a Multidimensional Parameter Space. *Advanced Energy Materials* **2025**, *n/a* (n/a), 2404957. https://doi.org/10.1002/aenm.202404957.

(5) Cakan, D. N.; Kumar, R. E.; Oberholtz, E.; Kodur, M.; Palmer, J. R.; Gupta, A.; Kaushal, K.; Vossler, H. M.; Fenning, D. P. PASCAL: The Perovskite Automated Spin Coat Assembly Line Accelerates Composition Screening in Triple-Halide Perovskite Alloys. *Digital Discovery* **2024**, *3* (6), 1236–1246. https://doi.org/10.1039/D4DD00075G.

(6) Sadeghi, S.; Bateni, F.; Kim, T.; Son, D. Y.; Bennett, J. A.; Orouji, N.; Punati, V. S.; Stark, C.; Cerra, T. D.; Awad, R.; Delgado-Licona, F.; Xu, J.; Mukhin, N.; Dickerson, H.; Reyes, K. G.; Abolhasani, M. Autonomous Nanomanufacturing of Lead-Free Metal Halide Perovskite Nanocrystals Using a Self-Driving Fluidic Lab. *Nanoscale* **2024**, *16* (2), 580–591. https://doi.org/10.1039/D3NR05034C.

(7) Wang, T.; Li, R.; Ardekani, H.; Serrano-Luján, L.; Wang, J.; Ramezani, M.; Wilmington, R.; Chauhan, M.; Epps, R. W.; Darabi, K.; Guo, B.; Sun, D.; Abolhasani, M.; Gundogdu, K.; Amassian, A. Sustainable Materials Acceleration Platform Reveals Stable and Efficient Wide-Bandgap Metal Halide Perovskite Alloys. *Matter* **2023**, *6* (9), 2963–2986. https://doi.org/10.1016/j.matt.2023.06.040.

(8) Zhang, J.; Hauch, J. A.; Brabec, C. J. Toward Self-Driven Autonomous Material and Device Acceleration Platforms (AMADAP) for Emerging Photovoltaics Technologies. *Acc. Chem. Res.* **2024**, *57* (9), 1434–1445. https://doi.org/10.1021/acs.accounts.4c00095.

(9) Li, J.; Li, J.; Liu, R.; Tu, Y.; Li, Y.; Cheng, J.; He, T.; Zhu, X. Autonomous Discovery of Optically Active Chiral Inorganic Perovskite Nanocrystals through an Intelligent Cloud Lab. *Nat Commun* **2020**, *11* (1), 2046. https://doi.org/10.1038/s41467-020-15728-5.
